# Engaging communities in therapeutics clinical research during pandemics: Experiences and lessons from the ACTIV COVID-19 therapeutics research initiative

**DOI:** 10.1017/cts.2024.561

**Published:** 2024-10-15

**Authors:** David A. Wohl, Stacey J. Adam, Kevin W. Gibbs, Ari L. Moskowitz, Thomas L. Ortel, Upinder Singh, Nikolaus Jilg, Teresa H. Evering, William A. Fischer, Babafemi O. Taiwo, Eric S. Daar, Christopher J. Lindsell, Susanna Naggie, Russell L. Rothman, Sarah E. Dunsmore, M. Patricia McAdams, Julia Vail, Dushyantha Jayaweera

**Affiliations:** 1 Institute of Global Health and Infectious Diseases, University of North Carolina, Chapel Hill, NC, USA; 2 Foundation for the National Institutes of Health, Bethesda, MD, USA; 3 Wake Forest University School of Medicine, Winston-Salem, NC, USA; 4 Montefiore Medical Center, New York, NY, USA; 5 Departments of Medicine and Pathology, Duke University, Durham, NC, USA; 6 Department of Medicine, Duke University, Durham, NC, USA; 7 Stanford University, Stanford, CA, USA; 8 Division of Infectious Diseases, Massachusetts General Hospital, and Division of Infectious Diseases, Brigham and Women’s Hospital, Harvard Medical School, Boston, MA, USA; 9 Weill Cornell Medicine, New York, NY, USA; 10 Division of Pulmonary Diseases and Critical Care Medicine, University of North Carolina, Chapel Hill, NC, USA; 11 Northwestern University, Chicago, IL, USA; 12 Division of HIV Medicine, Lundquist Institute at Harbor, University of California Los Angeles Medical Center, Los Angeles, CA, USA; 13 Department of Biostatistics and Bioinformatics, Duke University, Durham, NC, USA; 14 Vanderbilt University, Nashville, TN, USA; 15 National Center for Advancing Translational Sciences, National Institutes of Health, Bethesda, MD, USA; 16 Duke Clinical Research Institute, Durham, NC, USA; 17 Division of Infectious Diseases, Miller School of Medicine, University of Miami, Miami, FL, USA

**Keywords:** Community engagement, outreach, pandemics, outbreaks, COVID-19, antiviral treatment trials

## Abstract

This manuscript addresses a critical topic: navigating complexities of conducting clinical trials during a pandemic. Central to this discussion is engaging communities to ensure diverse participation. The manuscript elucidates deliberate strategies employed to recruit minority communities with poor social drivers of health for participation in COVID-19 trials. The paper adopts a descriptive approach, eschewing analysis of data-driven efficacy of these efforts, and instead provides a comprehensive account of strategies utilized. The Accelerate COVID-19 Treatment Interventions and Vaccines (ACTIV) public–private partnership launched early in the COVID-19 pandemic to develop clinical trials to advance SARS-CoV-2 treatments. In this paper, ACTIV investigators share challenges in conducting research during an evolving pandemic and approaches selected to engage communities when traditional strategies were infeasible. Lessons from this experience include importance of community representatives’ involvement early in study design and implementation and integration of well-developed public outreach and communication strategies with trial launch. Centralization and coordination of outreach will allow for efficient use of resources and the sharing of best practices. Insights gleaned from the ACTIV program, as outlined in this paper, shed light on effective strategies for involving communities in treatment trials amidst rapidly evolving public health emergencies. This underscores critical importance of community engagement initiatives well in advance of the pandemic.

## Introduction

A month after the World Health Organization (WHO) declared Coronavirus Disease 2019 (COVID-19) to be a Public Health Emergency (PHE) of International Concern, the National Institutes of Health (NIH) launched a public–private partnership to rapidly develop prophylactic and therapeutic interventions for COVID-19 [1,2]. The Accelerate COVID-19 Treatment Interventions and Vaccines (ACTIV) research initiative engaged governmental, industry, nonprofit, philanthropic, and academic partners to develop an infrastructure for the design and implementation of clinical trials that would directly respond to the fast-spreading COVID-19 pandemic. To advance the search for urgently needed therapeutics, ACTIV relied on a systematic process for identifying promising treatment candidates and then tasking protocol teams to rapidly develop and implement clinical trials to evaluate their safety and effectiveness [3,4].

Integral to the ACTIV mandate to expeditiously identify safe and effective therapeutic interventions for COVID-19 was the imperative to quickly enroll participants into the initiative’s trials. Each ACTIV treatment trial team devised tailored recruitment strategies tailored to their specific target populations, encompassing outpatients, inpatients, individuals with acute infections, and those in the convalescent phase. These tailored recruitment strategies were influenced by each unique trial design described in detail in the other papers in this issue. Each team aimed to recruit participants reflective of the pandemic and prioritized outreach to communities at disproportionate risk of both SARS-CoV-2 infection and severe COVID-19 and populations historically neglected and underrepresented in clinical research.

This paper provides an account of the approaches taken by ACTIV and its study protocol teams to achieve the overlapping goals of increasing domestic US public awareness about the trials, publicizing the studies to potential participants, and engaging communities most profoundly impacted by the pandemic. Highlighted are the challenges encountered by all ACTIV study teams, followed by a deeper dive into how the ACTIV-2 and ACTIV-6 outpatient trials approached community engagement, including those that were and were not overcome. As discussed below, limited data were collected on which to assess the effectiveness of the efforts taken by ACTIV and its therapeutic protocol teams to engage communities; however, by describing these approaches, as well as the lessons learned while implementing them, future research teams may be better able to prepare for and response to the next PHE.

## Overview of the ACTIV therapeutics trials reach

**Figure 1.**
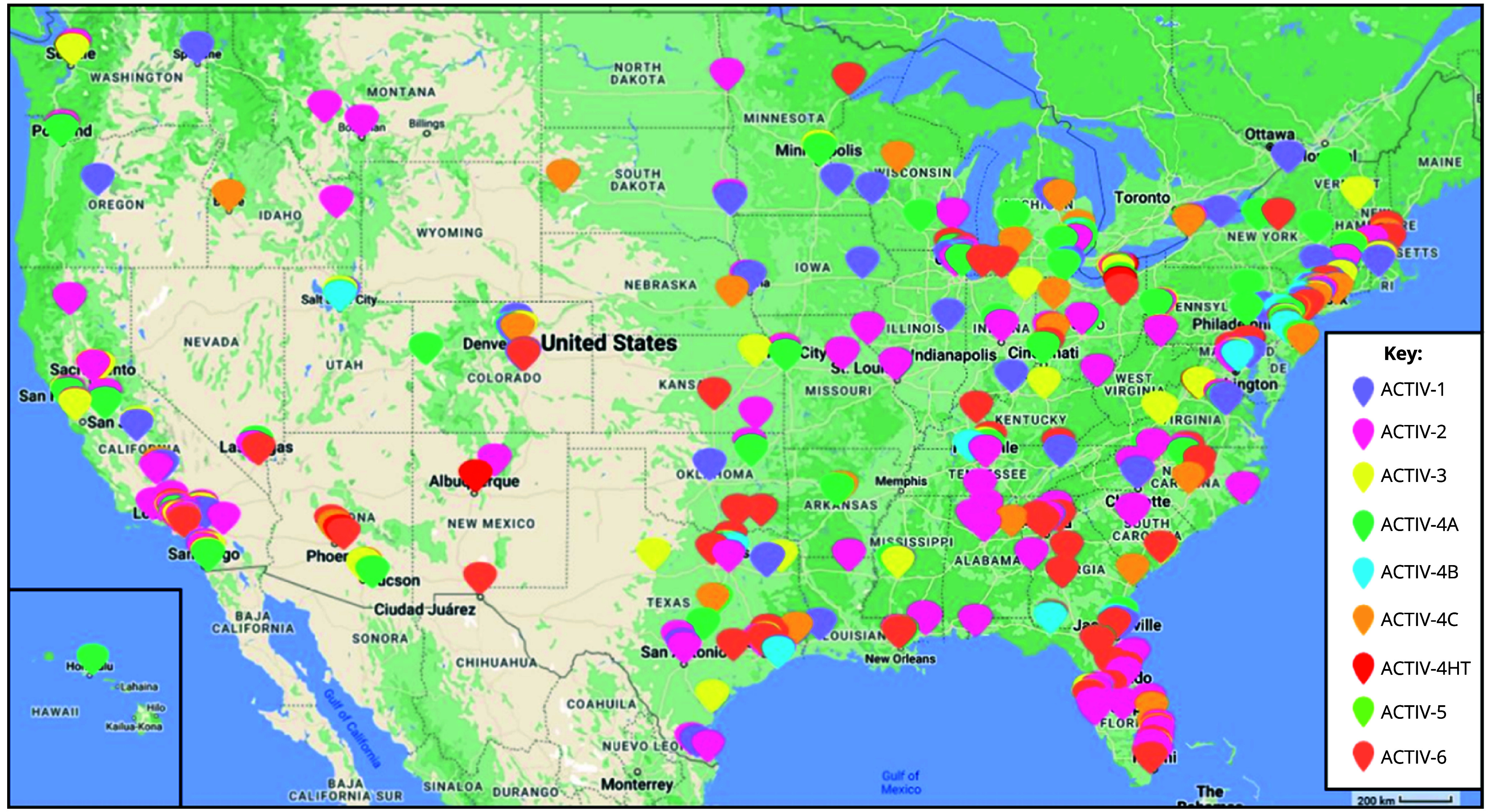
Map of ACTIV US sites. This map reflects the locations and geographic distribution of the sites for each of the ACTIV master protocol networks. It should be noted that some trials had deep site representation in areas of classically underserved communities, including those serving African American/Black, Hispanic/Latino, American Indian/Alaskan, and Hawaiian Native. While some trials did not have as deep of coverage, all ACTIV trial networks were very geographically diverse in the USA.

The ACTIV Therapeutic-Clinical Working Group developed research protocols to address different populations of patients with COVID-19 and therapeutic approaches [2,3]. ACTIV trials spanned phase II to III studies and enrolled either inpatients or outpatients (Fig. 1). Interventions tested included antivirals, immunomodulators, antithrombotics, and repurposed prescription medications. All but the ACTIV-4B, ACTIV-4C, ACTIV-5, and ACTIV-6 trials enrolled participants globally (Fig. 3). Trials shared some outreach tactics but many also developed unique recruitment and engagement assets, which are summarized in Table 1.

**Table 1.** Summary of ACTIV therapeutics master protocols recruitment strategies and geographic reach. The 11 ACTIV master protocols tested 37 single agents or combinations, and a summary of the trials and agents can be found in the ACTIV Overview report in this edition. In addition to the primary recruitment strategy for the trials, direct outreach to potential participants by the sites (i.e., data pulls of recent clinic visitors that broadly met inclusion criteria and phone (outpatient) and/or in person (inpatient) recruitment), this table summarizes the important information related to recruitment and geographic coverage of the trials, which helps explain the trials’ demographic makeup. However, unfortunately due to variable start and completion dates of the trials, nonuniform inclusion/exclusion criteria, differential routes of agent administration, and separate trial designs (traditional vs. decentralized), there are too many variables to attribute the variation in recruitment to only the variation in recruitment and outreach strategies. A more thorough comparison of methodologies would need to be tested in a more controlled experiment

Population	Master protocol	# of agents tested	Dates open for enrollment	Recruitment strategies	Overall enrollment (total participants)	# of sites^1^	Average participant enrollment/ site/month^1^	Geographic breakdown
**Inpatient studies**	**ACTIV-1**	3	Oct 15, 2020–Dec 30, 2021	Trial website and Combat COVID	1,971	85	1.5	29 states and District of Columbia 5 participating countries
	**ACTIV-3**	6	Aug 4, 2020–Dec 29, 2021	Trial website and Combat COVID	2,752	115	1.4	31 states and District of Columbia 15 participating countries
	**ACTIV-3B**	2	Aug 21, 2021–May 25, 2022	Trial websites and Combat COVID	473	28	1.9	31 states and District of Columbia (including sites for ACTIV-3) 2 participating countries
	**ACTIV-4A**	4	Sep 4, 2020–Mar 30, 2023	Trial website and Combat COVID	3,184	122	1.4	33 states 5 participating countries
	**ACTIV-4HT**	3	July 15, 2021–Sep 27, 2023	Trial website and Combat COVID	899	65	0.7	26 states 7 participating countries
	**ACTIV-5**	3	Oct 9, 2020–Feb 21, 2022	Trial website and Combat COVID	821	57	0.8	27 states 2 participating countries
**Outpatient studies**	**ACTIV-2/2D**	9	*ACTIV-2:* Aug 3, 2020–June 22, 2023 *ACTIV-2D:* July 29, 2023–Dec 8, 2023	Trial website, Combat COVID, call centers, paid search ads, virtual animated videos, social media, and newspaper ads	*ACTIV-2*: 4,044 *ACTIV-2D:* 2,081	*ACTIV-2:* 172 *ACTIV-2D:* 207	*ACTIV-2:* 1 ACTIV-2D: 0.7	*ACTIV-2* 37 states, the District of Columbia, Puerto Rico (including sites for ACTIV-2D) 7 participating countries *ACTIV-2D:* 37 states, the District of Columbia, Puerto Rico (including sites for ACTIV-2) 10 participating countries
	**ACTIV-4B**	3	Sep 7, 2020–Aug 5, 2021	Trial website and Combat COVID	657	50	1.2	15 states and District of Columbia
	**ACTIV-6**	7	July 21, 2021–April 29,2024	Trial website, Combat COVID, call centers, search ads, social media, and radio ads	10,956	110	3	28 states and US Wide Call Center *(Enrollment from all 50 states)*
	**ACTIV-4C (convalescent)**	1	Feb 9, 2021–June 23, 2022	Trial website and Combat COVID	1,217	106	0.7	26 states 5 participating countries

*Note:* ACTIV = Accelerating COVID-19 Therapeutic Interventions and Vaccines.

### Challenges to community engagement, outreach, and recruitment

When the ACTIV initiative was launched, major challenges to participant recruitment were clearly recognized (Table 2). Implementing clinical trials during an active pandemic of a respiratory virus required adequate staffing, training, and supplies of personal protective equipment, as well as the adoption of strict infection prevention and control procedures. In addition to these operational demands, unique challenges to community engagement were encountered across the studies.

**Table 2.** Challenges to recruitment to ACTIV COVID-19 therapeutics trials. Based on the opinion of the ACTIV investigators in the writing committees

Major challenges to recruitment to ACTIV COVID-19 therapeutics trials
Infection control requirements that limited clinical research operations including site access, exam space, face-to-face interactions, shortage of personal protection equipment,
Shortage of test tubes, swabs, COVID-19 testing equipment.
Mitigation policies prevented face-to-face stakeholder interactions
Widespread dissemination of misinformation about COVID-19 [5,7–13]
Popular belief in the efficacy of unproven interventions
Competition for participants with other treatment trials
Distrust of science and academic institutions by racial, ethnic, and other minority groups mistreated and neglected by healthcare and research
Limitations of web-based approaches to reach populations at risk of infection and adverse consequences of COVID-19 including older people and people of color
Changes in standard of care treatment options as the pandemic progressed
Traditional community engagement efforts, such as in-person meetings with community representatives and organizations, were not possible
An unprecedented number of investigational trials simultaneously attempting to enroll these patients
Many in-patients with severe COVID-19 were often unable to consent for trial enrollment themselves and challenges contacting the legally authorized representatives (LARs).
Concurrent enrollment in various trials and lack of coordination, driven by the pressing need for quick results during the pandemic, caused confusion among participants, researchers, and ethics committees to avoid competition, conflicts of interest, and therapeutic misconceptions.
Complexity of remote and electronic consent affecting people with poor social determinants of health

*Note:* ACTIV = Accelerating COVID-19 Therapeutic Interventions and Vaccines.

Traditional community engagement efforts, such as in-person meetings with community representatives and organizations, were not possible; therefore, novel strategies for public messaging and outreach, such as social media platforms, were needed. However, concerns existed that some digital strategies for engagement would not reach many of those most vulnerable to COVID-19, including older individuals.

During the initial phases of the pandemic, communities of color were disproportionately at risk of both SARS-CoV-2 infection and severe COVID-19 [5]. As such, there was awareness among researchers that messaging about the ACTIV studies had to reach and resonate with these communities and acknowledge and address mistrust of research justifiably present in many [6].

Recruitment to COVID-19 treatment trials also had to contend with widespread misinformation and growing cynicism regarding public health approaches to the pandemic and data from early prevalent trials being negative [5,7–13]. This required ACTIV study teams to invest not only in campaigns to advertise the trials but also to provide accurate information to the public and potential participants about COVID-19.

While many of these challenges were shared across the ACTIV trials, those recruiting inpatients faced unique pandemic-related challenges, which are now captured in Table 2.

Lastly, as described in other reports on this issue, despite the overarching goal to rapidly develop and implement COVID-19 protocols, application of master protocols, amalgamation of complex research processes across different large clinical research networks, and recruitment of both inpatients and outpatients to a variety of interventions, recruitment to some of the ACTIV trials was slower than planned. Enrollment in early industry and investigator-initiated trials was already underway when the first ACTIV trial opened, and some trials were enrolling at sites participating in ACTIV, leading to competition for participants. This concurrent enrollment in various trials and lack of coordination, driven by the pressing need for clinically applicable results during the PHE, caused confusion among participants, researchers, and ethics committees regarding avoiding competition, conflicts of interest, and therapeutic misconceptions.

### ACTIV therapeutics community engagement and outreach response

At the time of publication, more than 26,000 individuals had been enrolled in the 11 original ACTIV master protocols. Key engagement and outreach activities that supported recruitment of these participants are detailed below with recognition of different approaches taken by studies recruiting outpatients and inpatients (Table 3).

**Table 3.** ACTIV therapeutics community engagement and outreach response

ACTIV therapeutics community engagement and outreach response
Public messaging and research marketing
Websites, web portals, and call centers
Digital strategies: social media, paid search, display ads
Earned media
COVID-19 testing
Community outreach and engagement

*Note:* ACTIV = Accelerating COVID-19 Therapeutic Interventions and Vaccines.

### Public messaging and research marketing

As the ACTIV trials launched, each protocol team created strategies to support outreach and recruitment. At the most fundamental level, study teams sought to publicize the research to the public. In this, they were assisted by the Department of Health and Human Services (DHHS) and the NIH, which listed the ACTIV trials on frequently visited government COVID-19 websites. Leaders from both agencies recorded videos promoting ACTIV that were posted on the websites and circulated to employees.

However, it became evident a more active awareness strategy was needed to accelerate recruitment and study completion, such as that deployed by the COVID-19 vaccine trials. Just as the ACTIV trials were being launched in April 2020, SARS-CoV-2 vaccine trials developed by the federally funded COVID-19 Prevention Trials Network (CoVPN) were preparing to recruit and were supported by an engaging nationwide public and community outreach program, the centerpiece of which was a registry those interested in participating in upcoming trials could join [14]. This registry was buoyed by well-designed multimedia (e.g., social media, television, and radio) campaigns designed to highlight the CoVPN commitment to diversity and equity. Simultaneously, public health and political leaders underscored the critical importance of vaccines, which led to more attention being paid to vaccine trials. Consequently, there was a notable surge in public awareness and enthusiasm for these studies compared to trials aimed at developing effective treatments for COVID-19.

Unlike COVID-19 vaccine trials, which were able to cast a wide net to millions of eligible participants, COVID-19 treatment studies recruited from a much smaller number of individuals within days of being diagnosed with SARS-CoV-2 infection – people who were typically feeling ill, scared, confused by mix messaging, mistrust and with limited transportation options due to risk of viral spread. Therefore, ACTIV protocol teams recognized a need for broad awareness of these trials prior to a positive test, as well as those just diagnosed.

A boost to the effort to increase public awareness of the ACTIV trials came in December 2020 when the DHHS created a central website including information on all government-sponsored COVID-19 trials, both vaccine and therapeutic interventions (combatcovid.hhs.gov) [15]. (Supplemental Fig. 1) The DHHS Combat COVID communications campaign ultimately determined they connected and engaged with over 80 organizations across diverse participant groups. The outreach achieved over 3,500,000 Combat COVID interactions through webinars, e-blasts, social media, and website promotion. Overall, the team distributed 5,500 Combat COVID materials throughout local communities. As described below, the presence of the ACTIV initiative on governmental websites was augmented by web-based strategies developed by protocol teams that included study-specific websites, search engine marketing, and banner and social media ads.

Public awareness of ACTIV trials was also promoted using traditional media outlets. ACTIV-6, for example, produced radio ads, including streaming and broadcast played on Black-owned/led and Spanish-speaking channels to reach broad audiences, including rural communities who tend to spend more time driving than those in urban areas. ACTIV-2 placed print ads in Black-owned/led newspapers in several select markets where COVID-19 incidence was high and/or increasing and contracted a community-based organization to recruit in barbershops and salons in several communities of color to promote awareness where rates of COVID-19 were rising.

In addition to such large-scale national efforts, trial awareness was promoted locally as research sites took advantage of knowledge of COVID-19 epidemiology in their communities and appreciation of the sensitivities and priorities of those living in them. Sites leveraged relationships with community partners to disseminate messages about ACTIV trials and shared successful outreach approaches on regularly held conference calls attended by other sites. However, due to the nature of the contagion and being unprepared to face a pandemic of this magnitude, there was no time to set forth any comparisons between the different strategies mentioned above. As such, there is little incremental knowledge to use in the future without knowing whether or not these were successful. But this is a lesson itself to be learned in preparing for future pestilences.

### Websites, web portals, and call centers

Internet presence is now essential for most recruiting clinical trials; indeed, early in the PHE, the Internet was the only publicly available source of information about the ACTIV trials. The Combat COVID website was useful for promoting general awareness of existence of ACTIV trials and also served as a gateway for enrollment by linking to study-specific web pages. ACTIV trial websites were a public-facing resource for those interested in learning about the trials (Supplemental Fig. 2) [16,17]. This was particularly important to the trials enrolling outpatients, and the ACTIV-2 and ACTIV-6 websites provided study details in both English and Spanish, including rationale, design, and descriptions of interventions. Using graphics and videos, including animation and recordings made by investigators and others, they provided easy-to-understand messaging about the trials.

Importantly, these websites also served as significant portals for recruitment. Both the ACTIV-2 and ACTIV-6 websites featured downloadable recruitment resources. The ACTIV-2 website also had an animated video explaining the trial. Both trial websites included links to screening surveys to determine initial eligibility and were configured to connect potential participants to a 24-hour call center staffed by English- and Spanish-speaking representatives. Those passing initial screening were linked with a site near the caller – a “warm hand-off” that allowed for an efficient and seamless flow of study candidates from initial encounter with the website to an appointment for screening at a site. However, none of these were objectively evaluated due to pandemic conditions and limited resources.

ACTIV-6 used a call center, the Duke Clinical Research Institute-Participant Research Operations (DCRI-PRO), that potential participants could call to enroll in the trial directly or be connected to a site via an EDC platform.

Early on, both ACTIV-2 and ACTIV-6 formed community advisory committees to guide protocol teams, particularly in community engagement. Led within the National Institute of Allergy and Infectious Diseases (NIAID)-sponsored US AIDS Clinical Trials Group (ACTG), ACTIV-2 created a community advisory board (CAB) with a diverse membership by US region, gender identification, race, and ethnicity. The CAB contributed extensively to the study website, including its look and content, as well as to all other study outreach initiatives, advertisements, and recruiting materials. Similarly, ACTIV-6 created a stakeholder advisory committee (SAC) composed of physicians and patients/caregivers to guide uniform processes and procedures with an inclusive and patient-centered lens [18]. The SAC had substantive input into the study website, recruitment flyers, and medication packaging inserts (Supplemental Fig. 3).

### Digital strategies: Social media, paid search, display ads

While study websites contained detailed information about the ACTIV trials and were able to lead potential participants to call centers or directly to a study site, people needed to be drawn to these websites to make them effective. As mentioned, passive strategies such as including links to sites on heavily trafficked COVID-19 websites were used. More active strategies were also formulated to spread awareness. ACTIV-2 and ACTIV-6 created social media presences on the platform known at the time as Twitter and on Facebook and Instagram. Content developed with the community advisors focused on marketing of trials and included links to study-specific web pages. Suggested social media posts, including images, were shared with sites through social media kits, so they could disseminate information on their own institutional channels.

A standard digital marketing strategy is search engine marketing, also known as paid search, in which advertisers bid on keywords or phrases (e.g., COVID-19 medication and COVID-19 treatment study) to increase visibility in search results. Search engine marketing in English and Spanish was used by the Combat COVID team, ACTIV-2, and ACTIV-6, so those seeking information about COVID-19 treatments or trials using the Google search engine could receive a trial advert atop other results. In addition to writing ad content that resonates with people seeking COVID-19 treatment options, Combat COVID, ACTIV-2, and ACTIV-6-targeted ads in geographic areas that were or predicted to become hot spots for COVID-19 infection, including disproportionately impacted communities. In ACTIV-2, Spanish-paid search ads outpaced English ads, driving more traffic to the study website. Paid search was an effective channel in increasing the number of users who expressed interest in ACTIV-2 and ACTIV-6 by clicking the “Enroll Now” or “Click to Call” buttons on the websites.

Display ads are another search engine strategy used by the Combat COVID, ACTIV-2, and ACTIV-6 teams. These ads combine text, images, and a URL that links to the study website for more information. In ACTIV-6, display ads have successfully reached people identifying as Spanish through ads on mobile games. Display ads on news-related sites have been helpful in spreading awareness about ACTIV-6 as the news cycle changed during the pandemic. ACTIV-2 was placed on websites used by healthcare providers (e.g., WebMD).

For these outpatient ACTIV trials, digital outreach strategies were co-produced with community representatives. For ACTIV-2, all ads were developed with the CAB and maintained fidelity to the website look and messaging (Supplemental Fig. 2). All ad content in ACTIV-6 was developed with insights from the SAC and emphasized content and imagery that resonated with and reflected diverse and inclusive populations. For the Combat COVID website, ads and messaging were developed in conjunction and with consultation from relevant trial teams whose study information was included in the site. Ad content was also refreshed throughout the PHE to ensure it remained relevant and compelling to key audiences.

### Earned media

Unpaid publicity, also called earned media, is free, although more challenging to produce than digital media strategies. ACTIV leadership, protocol teams, and site investigators were encouraged to work with their institutional public relations teams to share study press releases and garner attention to the initiative. ACTIV-2 worked with a US-based international health media company to guide digital strategies and create a media tool kit for study sites and investigators to use to drive interest from news outlets and publicity about the initiative. The company also frequently contacted major English and Spanish media in different locales to solicit interest in covering ACTIV stories, leading to coverage by local and national radio (e.g., Keepin’ it Real with Rev. Al Sharpton radio show) and television programs (e.g., Telemundo’s Nuevo Dia program).

The ACTIV trial teams were diverse, and this facilitated community messaging. Protocol team members were often asked to speak with media, including in Spanish and other languages, and the team diversity was valuable in promoting research and providing factual information about COVID-19 to the public.

ACTIV-2 and ACTIV-6 also supported engagement with media by providing site media tool kit talking points, as well as a press release template. ACTIV-6 employed a robust media outreach strategy for returning study results, most notably for study arms focusing on ivermectin. Considering public attention to and misinformation around this medication, there was an urgent need to communicate findings quickly and accurately to inform treatment. The ACTIV-6 Clinical Coordinating Center at DCRI coordinated with the Duke Health News Office to develop and distribute a press release linking to the pre-print of the ivermectin results, earning coverage in the *New York Times, Forbes,* and other prominent media outlets. ACTIV-6 also produced supplementary materials including plain-language results summaries to facilitate the sharing of study findings.

Communications teams from NIH, the Foundation for the National Institutes of Health (FNIH), and industry partners also worked to provide updates and press releases around ACTIV trial results on their websites and social media channels. These announcements were often picked up by national news outlets, as well as scientific trade press sources, such as *GenomeWeb, STAT, FierceBiotech*, and others.

### COVID-19 testing

Until home testing became widely available, testing for SARS-CoV-2 infection was done at clinics, hospitals, or dedicated testing centers. COVID-19 testing centers offered an opportunity to connect with individuals testing positive for SARS-CoV-2 and potential candidates for ACTIV outpatient trials. However, as testing sites were established by thousands of different organizations, negotiating on-site recruitment activities was a challenge. As such, some enterprising ACTIV sites established their own COVID-19 testing operations to support recruitment. Sites also collaborated with emergency and urgent care centers to solicit interest in participation among patients diagnosed with COVID-19. Others worked within centralized healthcare systems to make recruitment materials available at their COVID-19 testing centers and at emergency and urgent care centers. This approach diminished with the advent of home testing kits.

Several ACTIV-2 and ACTIV-6 sites used their institution’s electronic medical record (EMR) systems to flag patients with a positive COVID-19 test. These patients were then contacted via the EMR or phone to explore interest in study participating in the study.

### Community outreach and engagement

**Figure 2.**
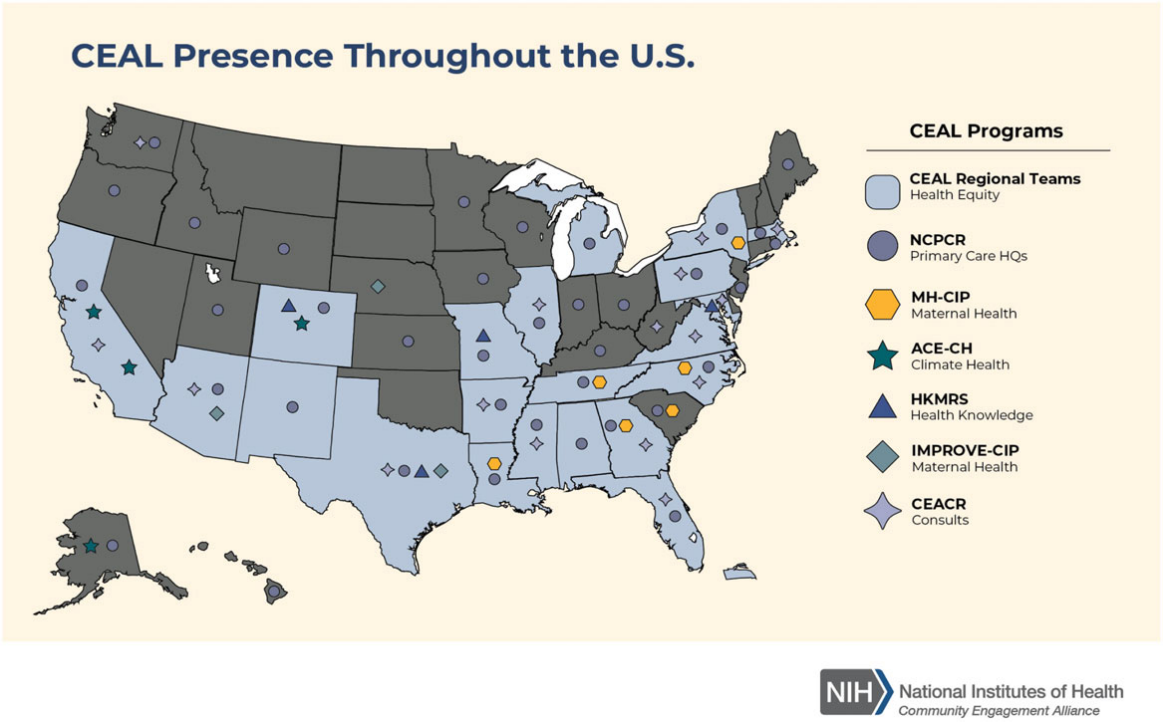
National Institutes of Health Community Engagement Alliance (CEAL) footprint within the USA. This is the current presence of all of the CEAL activities. Map is from this website: https://nihceal.org/about-community-engaged-research-and-ceal, where more information can be found about CEAL efforts. For purposes of reference, only the original CEAL Regional Teams were present and assisting ACTIV during much of the pandemic. CEAL = Community Engagement Alliance, NIH = National Institutes of Health.

Digital and other publicizing efforts aimed to increase awareness of ACTIV trials; however, it was also critical to promote public acceptance of the research. Many ACTIV trial teams collaborated with the NIH’s Community Engagement Alliance (CEAL) Against COVID-19 Disparities teams [19]. CEAL was established to lead outreach and engagement efforts in underserved ethnic and racial minority communities disproportionately affected by the COVID-19 pandemic, specifically African Americans/Blacks, Hispanic/Latinos, American Indians/Alaskan Natives, Asian Americans, and Native Hawaiians and Pacific Islanders. The goals of CEAL to establish partnerships with these communities, address misinformation within communities of color, grow an understanding and trust in science, and accelerate the uptake of beneficial treatments aligned well with those of ACTIV. During the COVID-19 pandemic, CEAL teams deployed a set of public messages presenting accurate information about COVID-19, created mechanisms, processes, and structures to conduct urgent community-engaged research and outreach, and used these tools to support and expand community outreach efforts by other NIH COVID-19 research efforts, such as ACTIV, CoVPN, and Rapid Acceleration of Diagnostics (RADx). CEAL teams were formed in geographies across the USA (Fig. 2). ACTIV-6 utilized CEAL’s Community Engagement Alliance Consultative Resource – initially to obtain advice on recruitment strategies and more recently to obtain recommendations for a final participant thank you when the ACTIV-6 platform completes final enrollment.

Domestically, ACTIV teams with and independent of CEAL worked to reach out to “super” stakeholders, individuals, or organizations with substantial influence to make them aware of the work, obtain advice and feedback, and request assistance with outreach. Such super-stakeholders included large advocacy organizations representing interests of older persons, African Americans/Blacks, Hispanic/Latinos, American Indians/Alaska Natives, Asian Americans, immigrants, and others. Leaders from federally qualified, community healthcare systems were engaged, as were major health professional organizations, including those representing minority healthcare workers and health insurance companies. Study teams also conferred with tribal governments and council members to develop strategies for bringing ACTIV studies to areas where American Indians could participate.

ACTIV-2 also contracted with a well-known African American-owned community-based organization in Los Angeles, founded to address racial health inequities, to develop a strategic plan to support trial participation by persons of color across the USA. This work led to establishment of the abovementioned multi-city barbershop and salon campaign in which the organization’s staff conducted training to prepare shop workers to discuss ACTIV-2 with patrons and disseminate recruitment materials. Leaders also met with Black political, religious, and media leaders in these cities as part of a comprehensive program of bidirectional dialog.

While traditional methods for community engagement such as face-to-face town halls and local stakeholder group meetings were largely impossible early in the pandemic, online forums were often used to encourage community dialog. Teleconferencing and social platforms allowed study teams to regularly hold events where many attendees could learn about the research, ask questions, and provide feedback. Such events could be national, as were those held for staff at federally qualified and community health centers, or local, organized in conjunction with community partners.

ACTIV-2 and ACTIV-6 also sought to open at sites accessible to communities of color, including people identifying as African American/Black, Hispanic/Latino, and American Indian/Alaska Native. Sixty percent of ACTIV-6 sites were located in counties where the African American/Black population is above the US average and 50% of ACTIV-6 sites were located in counties where the Hispanic/Latino population is above the US average. ACTIV-6 also engaged with the networks for Clinical and Translational Research in Institutional Development Award (IdeA) states which have historically received the lowest amount of NIH funding and often contain rural, underserved, and unique racial/ethnic populations. Representatives from the ACTIV-6 operations and communications teams met with investigators and communications specialists from institutions in the IdeA Clinical and Translational Research Network, so they could be involved with ACTIV-6 as enrollment sites and/or communications partners. One example of these interactions was translation of the ACTIV-6 study brochure and recruitment flyer into Hawaiian, Samoan, and Tagalog based on the request of an IdeA investigator from the University of Hawaii.

ACTIV-2 worked with the ACTG and the NIH Tribal Health Research Office to organize meetings with American Indian governing bodies to introduce and receive feedback about this trial and identify and support research sites, including clinics with little prior research experience, located on or near reservations. Town halls with American Indian advocacy groups were also conducted to identify unique challenges to recruitment, such as concerns regarding the disposition of blood and other biosamples collected during the research, and brainstorm potential solutions to address these issues proactively.

### Inpatient trial participant recruitment

Strategies developed for community outreach and engagement for ACTIV outpatient trials differ in many ways from those required for ACTIV inpatient studies. The success of ACTIV-1, -3, -3B, -4A, and -5 was dependent on recruitment of patients in the hospital, including in emergency departments, who had tested positive for COVID-19. As such, a major focus was placed on reaching out to and eliciting support from hospital clinicians caring for COVID-19 patients to make them aware of these studies. In addition, priority was placed on developing easily understandable and accessible materials for participants and families, caregivers, and legally authorized representatives (LARs) to learn about the trials. Overall, these efforts included:

- Frequent review of SARS-CoV-2 test results to identify hospitalized patients with positive test results.
- Distribution of template memos and slide presentations to colleagues treating COVID-19 patients describing the trials and providing contacts.
- Posting of study posters/flyers for some trials strategically throughout the hospital.
- Provision of study overview videos and flipbooks, in the case of ACTIV-3 and -3B, in English and Spanish to be shared with potential participants and their families, caregivers, and LARs.

For inpatient trials, study-specific ACTIV webpages in English and Spanish were hosted on the Combat COVID website, which also provided information about therapies and a search function for locations of facilities providing treatments for COVID-19. While it was recognized it was unlikely hospitalized patients with COVID-19 would search the Internet for relevant clinical trials, site staff often sent links to study-specific webpages for inpatient studies to families/caregivers/LARs, so they could read about the study (for further information on the specific practices for ACTIV-3/-3B and exemplar for the ACTIV trials refer to the Practical Application of Good Participatory Practices for Trials of Emerging Pathogen in this issue).

**Figure 3.**
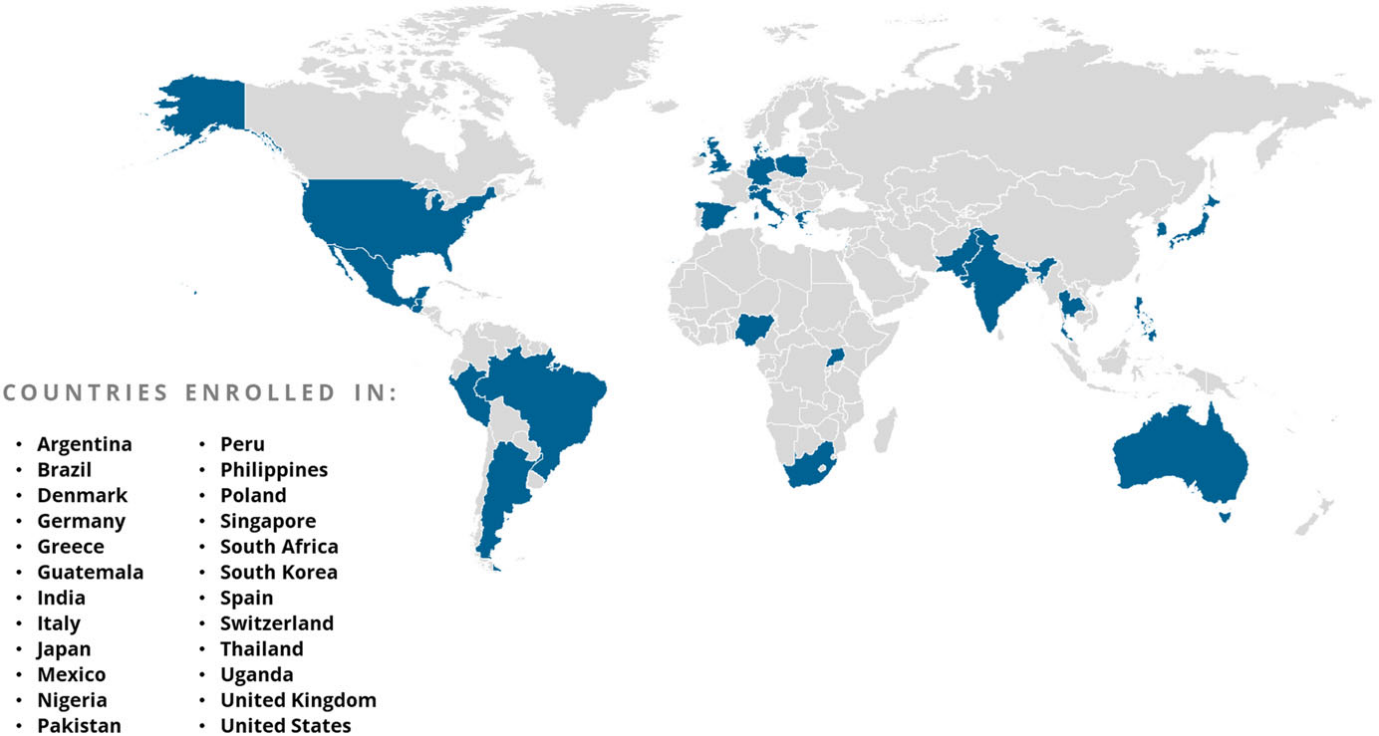
Countries participating in ACTIV trial enrollment globally.

Finally, a key element of success of inpatient ACTIV trials was leveraging of existing research networks and communities. Existing networks allowed for rapid integration and management of numerous COVID-19 trials into a complex and rapidly shifting pandemic ecosystem (Fig. 3). The community of investigators within existing networks also allowed for rapid troubleshooting and identification of novel practices to enhance ACTIV trial conduct. Success of leveraging existing trials networks highlights the importance of not only maintaining but also expanding trial infrastructure to prepare for future pandemics. Trial leaders should consider designs that allow for participation of hospitals with minimal research experience to ensure maximal efficiency and representativeness of trial populations.

### Communications and outreach strategies must be developed in parallel with protocols

The Combat COVID website, an attempt to centralize communications about COVID-19 and government-supported COVID-19 clinical trials, launched in December 2020 – 8 months after the launch of ACTIV and 4 months after the first ACTIV protocols opened for enrollment – leading to a delay in public outreach. The initial ACTIV trials created their own communications and outreach strategies without dedicated centralized communications team support. A lesson learned is that centralized communications support should start simultaneously with protocol development, so public awareness of upcoming trials and culturally appropriate materials for community engagement can be provided to trial teams, sites, community partners, and other stakeholders in a timely manner – ideally several weeks before a clinical trial protocol enrollment opens. Centralized communications support is critical when misinformation may be prevalent and easily accessible through popular media outlets.

### Advantages and disadvantages of team-based engagement efforts

As described above, multifaceted, innovative strategies were developed and implemented by ACTIV study teams to promote community engagement and recruitment of participants reflective of the COVID-19 pandemic. Many of these efforts successfully served individual studies and rooting these efforts at the team level fostered creative approaches tailored to the individual study. However, a disadvantage of this decentralized approach was lack of a comprehensive and coordinated overarching community engagement strategy including a shared vision, a set of well-articulated objectives for outreach and engagement across all of ACTIV, and the collection of metrics of success for selected approaches. The team-based approach also missed opportunities for protocol teams to pool resources to advance all trials and share best practices. Future research initiatives would benefit from a more unified and coordinated engagement planning, co-produced by investigators and stakeholders, providing a shared roadmap for raising awareness and cultivating acceptance. Such coordinated efforts need to be bolstered by an overarching system for evaluation that would collect metrics to assess the effectiveness of the activities undertaken.

### Community involvement should begin early

It was the experience of the ACTIV outpatient trials that the involvement of was community advisors was critical in shaping trial implementation and the design of community engagement activities. Community advisors for both ACTIV-2 and ACTIV-6 often took the lead in engagement and recruitment efforts. However, stakeholder engagement came relatively late after the ACTIV initiative was formulated and protocols drafted. In essence, leaders from highly impacted populations and communities were invited to the table only after protocols were “baked.” Having such stakeholders involved during the formative stages of the research program could have facilitated greater community buy-in, investment in, and acceptance of the research. Future efforts must include relevant, early representation from populations targeted to participate. As discussed below, such community engagement must start before rather than in the midst of a PHE.

In addition, large-scale outreach received outsized attention relative to efforts undertaken at the site level. Most sites were active in leveraging existing relationships to promote studies. Some established their own COVID-19 testing operations and successfully recruited those who tested positive. Sites were also often a source of information during the pandemic, and many conducted virtual education events to provide updated COVID-19 information, building goodwill and trust. These activities need to be supported with dedicated funding directed to sites.

### Use web-based approaches equitably

Given the challenges to outreach and engagement detailed above, reliance on web-based approaches was heavy. The rapid establishment of a strong web presence of ACTIV trials was an essential lynchpin for all other awareness, outreach, and engagement efforts. Well-designed and informative study websites also became the narrow part of an outreach funnel fed proximally by digital and other marketing and unpaid publicity. As described above, in some cases, study websites led to direct recruitment, facilitating enrollment for participants and sites. During the pandemic, when the Internet was relied on as the primary source of information regarding COVID-19, paid search on Google performed well in directing people to study websites with Google Spanish proving particularly effective based on web-traffic data. In general, social media was also seen as being useful as a means of increasing outpatient trial awareness and for directing potential participants to study websites and/or call centers.

However, there are inherent limitations to web-based strategies such as paid search, search ads, and social media campaigns including limited reach to many older individuals, particularly older individuals of color [20,21]. There exists a digital divide in the USA in which African American/Black access to the Internet has lagged behind that of people who are White [22,23,24]. While this gap has narrowed, there continue to be significant disparities in home broadband connectivity and racial and ethnic differences in Internet use frequency and duration. Such differences also exist for others, including Hispanics/Latinos, American Indians, and people living in rural areas or poverty – again, with older people less likely to be reached by these technologies [22]. These factors may have contributed to lower than desired proportion of ACTIV trial participants who were African American/Black or American Indian. Web-based awareness and recruitment approaches must address the digital divide. Partnering with trusted stakeholders and institutions who can promote research using their web-based communications may be a more effective approach, but more work needs to be done exploring how best to use the web to message to older people and people of color [20].

### Use traditional (non-web) media to reach those less connected

Paid advertisements in non-web-based media were also used to reach interested participants who were less likely to encounter an ad on social media or a search engine. ACTIV-6’s robust digital, social media, and radio campaign was critical to successful trial recruitment. As research suggests, healthcare providers are the most trusted source of information about clinical trials and the preferred first source for most people [25]. Advertising directed to healthcare providers was included in the ACTIV trial outreach strategy, both for inpatients and outpatients. Therefore, although web-based messaging can be an effective and economical approach, information should also be disseminated in non-web-based media.

Earned media is an important but under-utilized tool for increasing awareness of research initiatives. In ACTIV, federal partners issued press releases, but publicity efforts were largely left to site institutions and had to compete with other stories being pushed to media outlets. Contracting a health media company for ACTIV-2 greatly aided in garnering opportunities for investigators to speak to radio, television, and print media (in English and Spanish) and serves a model for future research initiatives.

### Participant diversity needs to be a goal

While collectively, ACTIV trial participants were diverse and enrollment exceeded standard clinical trial recruitment of critical populations as monitored by the FDA prior to the pandemic, demographic characteristics of participants varied by trial, with some more reflective of characteristics of the US pandemic than others (Table 4). This variability in representation of those most impacted likely reflects the above-mentioned challenges, as well as different approaches to outreach by each trial.

**Table 4.** ACTIV trial demographics. These trial demographics represent the final trial demographics for all ACTIV master protocols except, ACTIV-2D and ACTIV-4HT, who did not have their final by the time of submission. The demographics also are inclusive of global trial populations for those trials that recruited both in the USA and outside the USA (see Fig. 4 for global distribution of ACTIV trials.) The trial demographics show that while the ACTIV trials did succeed in recruiting populations that are more diverse that most therapeutic trials submitted to the FDA pre-pandemic, not all trials achieved full representativeness of the populations infected with COVID-19

Population	Master protocol	Sex, *n* (%)	Race n (%)							Ethnicity n (%)
		Female	White	Black/ African American	Asian	Native Hawaiian /Pacific Islander	American Indian/ Alaska Native	2 or more races	Other	Hispanic/ Latino Ethnicity
**Inpatient studies**	**ACTIV-1**	1125 (39.6)	1766 (62.3)	404 (14.2)	85 (3)	2 (0.06)	27 (1.0)	11 (0.4)	1305 (13.8)	1305 (46.03)
	**ACTIV-3**	1300 (42.4)	2,089 (68.2)	707 (23.0)	138 (4.5)	15 (<1)	12 (<1)	15 (<1)	87 (2.8)	570 (18.6)
	**ACTIV-3B**	183 (39)	340 (72)	73 (15)	17 (4)	6 (1)	7 (1)	0 (0)	28 (6)	121 (26)
	**ACTIV-4A**	1284 (39.4)	1934 (59.4)	593 (18.2)	132 (4.1)	20 (0.6)	50 (1.5)	14 (0.4)	136 (4.2)	990 (30.4)
	**ACTIV-4HT**	265 (41.8)	420 (66.3)	104 (16.4)	12 (1.9)	1 (<1)	9 (1.4)	1 (<1)	86 (13.5)	99 (16.6)
	**ACTIV-5**	297 (36.2)	578 (70.5)	151 (18.4)	34 (4.1)	2 (0.2)	4 (0.5)	2 (0.2)	25 (3.0)	151 (18.4)
**Outpatient studies**	**ACTIV-2/2D**	2138 (53)	3258 (81)	393 (10)	186 (5)	8 (0)	34 (1)	34 (1)	101 (3)	1773 (44)
	**ACTIV-4B**	388 (59.0)	494 (75.2)	78 (11.9)	9 (1.7)	4 (0.6)	3 (0.4)	0 (0)	27 (4.1)	178 (27.1)
	**ACTIV-6**	6735 (61.5)	8219 (75.0)	1077 (9.8)	405 (3.7)	17 (0.2)	56 (0.5)	201 (1.8)	917 (8.4)	3707 (33.8)
	**ACTIV-4C (convalescent)**	614 (50.5)	713 (58.6)	322 (26.5)	22 (1.8)	7 (0.6)	10 (0.8)	8 (0.7)	44 (3.6)	203 (16.7)

In addition to digital divide issues that may have led to less than equitable web-based messaging, diversity among the participants in the ACTIV trials may also reflect other shortcomings. Efforts were made to establish research sites in communities disproportionately impacted by COVID-19, including those that were predominately African America/Black, Hispanic/Latinx, and American Indian/Alaska and Hawaiian Native. However, this was likely insufficient and unable to overcome other barriers to participation that led to the underrepresentation of these and others in clinical research. A focus of the ACTIV community engagement was on access, and there was generally less attention paid to addressing entrenched distrust of biomedical research among African American/Black, American Indian, and other people in the USA who have been neglected and mistreated in research and healthcare – a mistrust aggravated by COVID-19 misinformation and disinformation [26].

A major lesson learned from the ACTIV trials experience is that the best time to prepare for a future PHE is before it happens. This is very relevant when recruiting individuals who are reflective of those at risk. Efforts to reach out to populations of color during ACTIV were made in the context of a rapidly evolving and frightening pandemic – a situation not ideal for establishing new trusted partnerships. It is during in-between periods when building these bridges to connect researchers and community needs to happen. Research networks and clinical investigators should regularly engage with communities in preparation of a PHE and together develop engagement plans that can be rapidly implemented, when needed. While several community engagement programs within the NIH and NIH-funded organizations have established such community relationships, insufficient coordination exists between these groups, diluting power and potential to be effective during the next crisis.

Community advisors to the ACTIV therapeutics trial teams often shared how greater diversity of the ACTIV protocol team members likely would have also benefited these trials. In addition to being morally just and equitable, inclusion of researchers with diverse backgrounds and identities can foster community trust and enthusiasm for the research. For example, contributions of African American scientists to COVID-19 vaccine development was highlighted by the NIH in its efforts to promote vaccination [27].

Earlier partnerships with the NIH-funded CEAL and other entities with aligned interests in eliminating disparities in research by providing equitable opportunities for study participation may also have benefited ACTIV in that these groups were building relationships with communities across the USA and could facilitate dialog regarding the research.

It is notable that Hispanic/Latino recruitment in ACTIV outpatient studies was high, as it was in most COVID-19 therapeutics trials. Hispanic/Latino representation in ACTIV trials may be a consequence of a high COVID-19 rate in this population across the USA and selection of high-enrolling sites with a predominantly Hispanic/Latino population (Fig. 1). As mentioned, some web-based outreach efforts in Spanish appeared to perform well, but there may be other factors, including sociological and cultural, to be explored further for lessons that might transfer to outreach to other populations.

### Delays impact community engagement

With delays that occurred in opening the ACTIV trials, community engagement and other supportive efforts often took a back seat to other study implementation priorities. For example, ACTIV-6 launched later in the pandemic and building effective relationships with sites, stakeholders, and communities took time. Study team members had to balance aggressive timelines for building the EDC system and opening the platform for enrollment with community engagement and stakeholder meetings. When ACTIV-6 did begin to enroll participants in June 2021, many sites and community organizations were overwhelmed and burnt out from previous participation in vaccine and other therapeutic clinical trials. Sites and community organizations particularly in resource-limited areas did not have bandwidth to support outreach and recruitment efforts for multiple COVID-19 trials over the 3-year time frame of the PHE.

Launch of interventional research studies during an outbreak is always extremely challenging. Barriers ACTIV faced in opening its trials should be studied, as should the ability of other trials (e.g., CoVPN in the USA) to be operating and enrolling relatively early. Learning how future clinical research responses can be nimbler and timelier will facilitate community engagement.

## Conclusions

**Figure 4.**
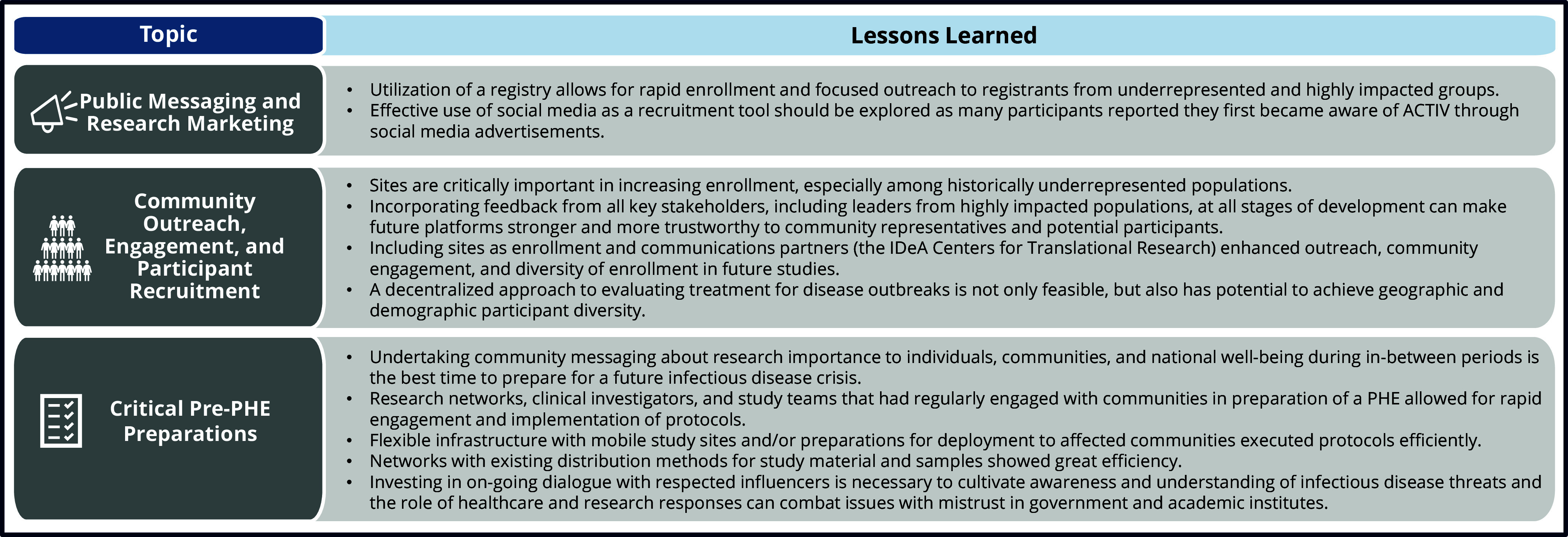
Overarching lessons learned from ACTIV engagement and recruitment activities applicable to future public emergencies. IDeA = Institutional Development Award; PHE = Public Health Emergency.

Design and implementation of the ACTIV trials during the pandemic highlight the considerable coordinated effort that made this, the US government’s flagship initiative to identify effective and safe treatments for COVID-19, successful. A variety of methods were used to message about the ACTIV therapeutics trials, and while there is an absence of data on their individual or collective effectiveness, the options chosen to address challenges to community engagement during a PHE offer insights for future research responses to emerging outbreaks. Similarly, the lessons the ACTIV researchers have taken from their experience are informative for preparation of therapeutics research responses for the next outbreak – preparation that must begin before the first case is detected and that is strengthened when investigators and the community share knowledge, build trust, and co-produce research. Lessons learned and recommendations put forward by the authors for participant engagement and recruitment for the next PHE are summarized in Table 5 (Recommendations) and Fig. 4 (Lessons Learned).

**Table 5.** Recommendations for participant outreach, engagement, and recruitment for the next Public Health Emergency

Recommendations for participant outreach, engagement, and recruitment for the next Public Health Emergency
Communications and outreach strategies must be developed in parallel with protocols
Advantages and disadvantages of team-based engagement efforts
Efforts must intensify to regain trust of communities among whom it has been chronically deficient and community involvement should begin early on research efforts during the next PHE
Use web-based approaches equitably
Use traditional (non-web) media to reach those less connected
Use traditional (non-web) media to reach those less connected
Participant diversity needs to be a goal
Delays impact community engagement

*Note:* PHE = Public Health Emergency.

## Supporting information

Wohl et al. supplementary material 1 Wohl et al. supplementary material

Wohl et al. supplementary material 2 Wohl et al. supplementary material

Wohl et al. supplementary material 3 Wohl et al. supplementary material

## Supplementary material

The supplementary material for this article can be found at https://doi.org/10.1017/cts.2024.561.
