# Supplementary figures and images for: Engaging communities in therapeutics clinical research during pandemics: Experiences and lessons from the ACTIV COVID-19 therapeutics research initiative
Source: J Clin Transl Sci. 2024 Oct 15;8(1):e156. doi: 10.1017/cts.2024.561 (PMC11557280; doi:10.1017/cts.2024.561)

## Slide 1
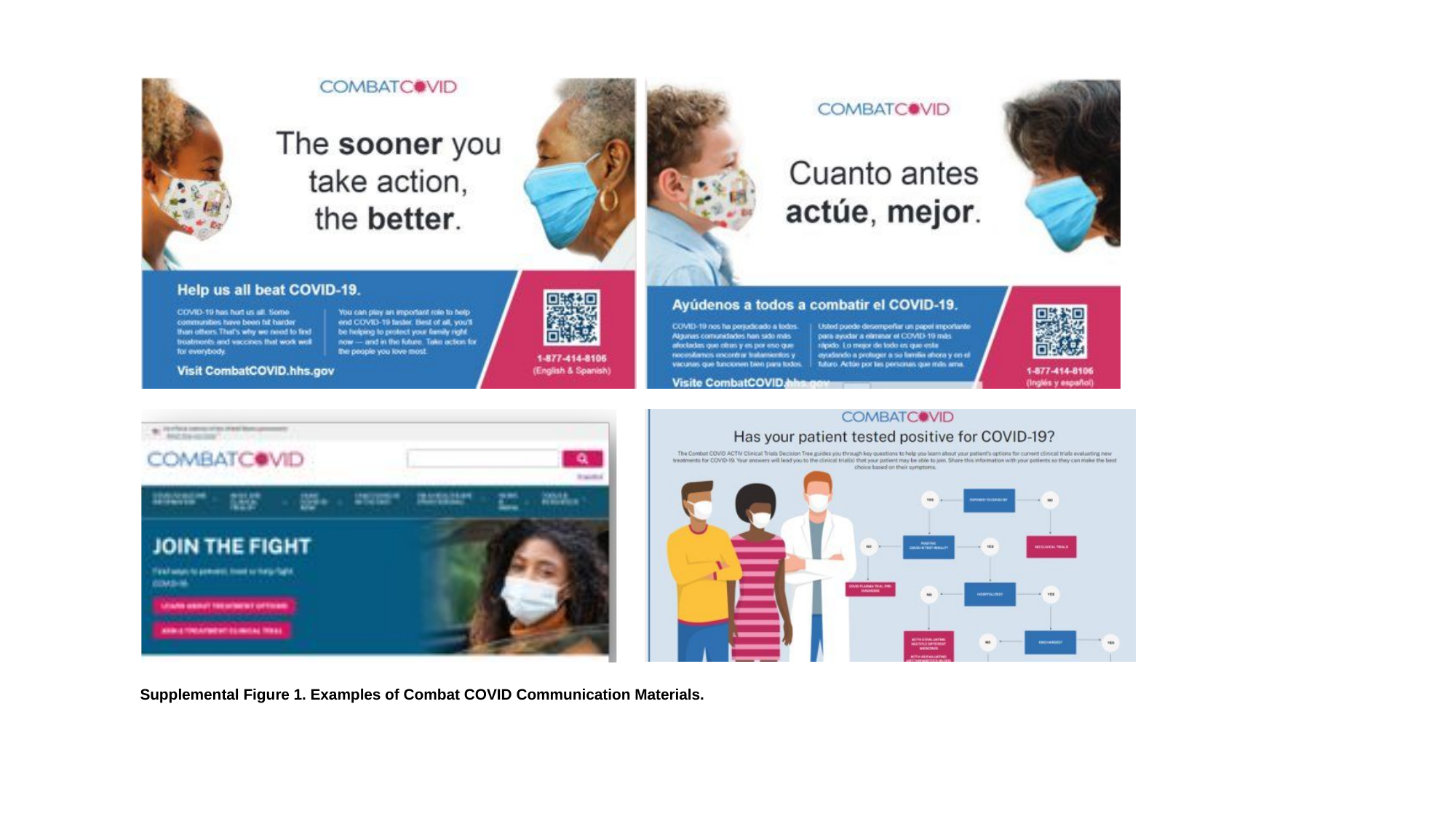

Supplemental Figure 1. Examples of Combat COVID Communication Materials.

Supplement: Wohl et al. supplementary material 1 — Wohl et al. supplementary material [file S2059866124005612sup001.pptx]

## Slide 1
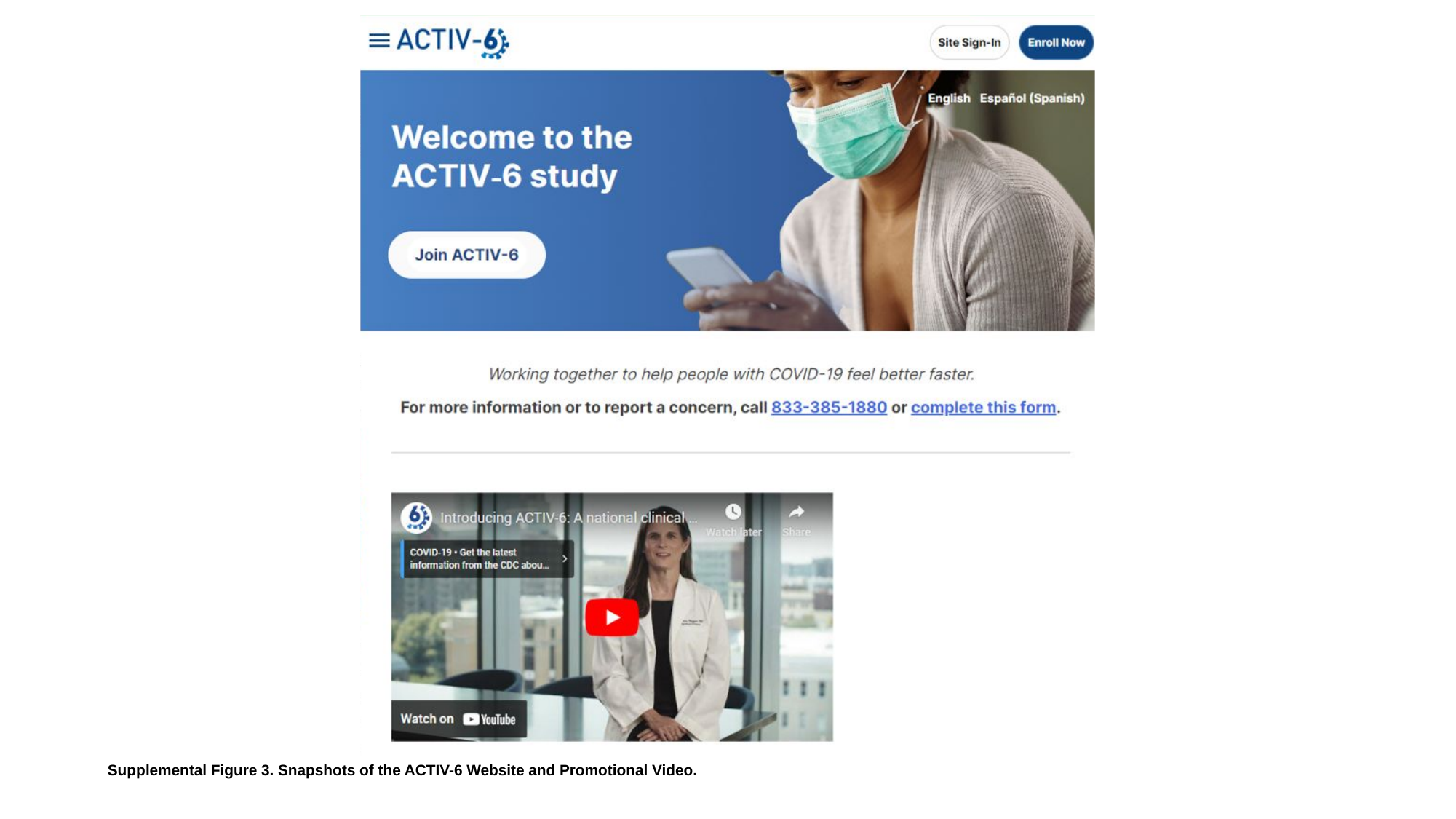

Supplemental Figure 3. Snapshots of the ACTIV-6 Website and Promotional Video.

Supplement: Wohl et al. supplementary material 3 — Wohl et al. supplementary material [file S2059866124005612sup003.pptx]
