# Supplementary material for: Engaging communities in therapeutics clinical research during pandemics: Experiences and lessons from the ACTIV COVID-19 therapeutics research initiative
Source: J Clin Transl Sci. 2024 Oct 15;8(1):e156. doi: 10.1017/cts.2024.561 (PMC11557280; doi:10.1017/cts.2024.561)
Supplement: Wohl et al. supplementary material 2 — Wohl et al. supplementary material [file S2059866124005612sup002.pptx]

## Slide 1
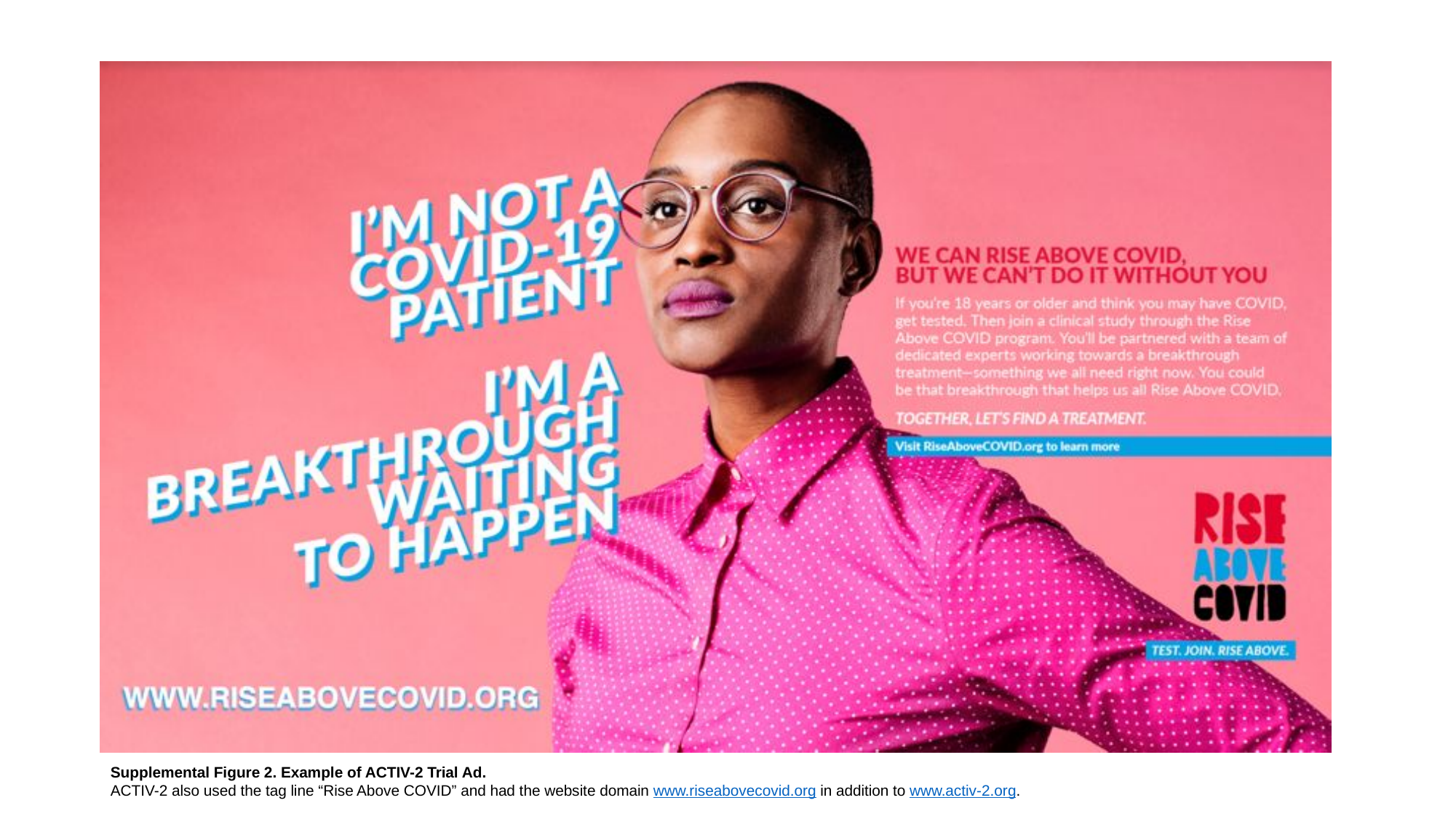

Supplemental Figure 2. Example of ACTIV-2 Trial Ad.
ACTIV-2 also used the tag line “Rise Above COVID” and had the website domain www.riseabovecovid.org in addition to www.activ-2.org.
